# Patterns of Brucellosis Infection Symptoms in Azerbaijan: A Latent Class Cluster Analysis

**DOI:** 10.1155/2014/593873

**Published:** 2014-12-14

**Authors:** Rita Ismayilova, Emilya Nasirova, Colleen Hanou, Robert G. Rivard, Christian T. Bautista

**Affiliations:** ^1^Republican Anti-Plague Station, Baku, Azerbaijan; ^2^Walter Reed Army Institute of Research, Silver Spring, MD 20910, USA; ^3^U.S. Army Medical Research Institute of Infectious Diseases, Fort Detrick, Frederick, MD 21702, USA

## Abstract

Brucellosis infection is a multisystem disease, with a broad spectrum of symptoms. We investigated the existence of clusters of infected patients according to their clinical presentation. Using national surveillance data from the Electronic-Integrated Disease Surveillance System, we applied a latent class cluster (LCC) analysis on symptoms to determine clusters of brucellosis cases. A total of 454 cases reported between July 2011 and July 2013 were analyzed. LCC identified a two-cluster model and the Vuong-Lo-Mendell-Rubin likelihood ratio supported the cluster model. Brucellosis cases in the second cluster (19%) reported higher percentages of poly-lymphadenopathy, hepatomegaly, arthritis, myositis, and neuritis and changes in liver function tests compared to cases of the first cluster. Patients in the second cluster had a severe brucellosis disease course and were associated with longer delay in seeking medical attention. Moreover, most of them were from Beylagan, a region focused on sheep and goat livestock production in south-central Azerbaijan. Patients in cluster 2 accounted for one-quarter of brucellosis cases and had a more severe clinical presentation. Delay in seeking medical care may explain severe illness. Future work needs to determine the factors that influence brucellosis case seeking and identify brucellosis species, particularly among cases from Beylagan.

## 1. Introduction

Brucellosis is a contagious bacterial disease among humans and usually presents as an acute febrile infection with nonspecific flu-like symptoms such as fever, headache, back pain, malaise, and night sweats [1, 2]. Brucellosis is endemic in Azerbaijan, with the first case reported in 1922. Azerbaijan's population of 9.6 million is evenly split between urban and rural residents, with almost 40% of the labor force employed in the agricultural or livestock industry, including raising cattle, sheep, and goats [3]. Between 1995 and 2009, 7,983 brucellosis cases were reported, with an average of 300–400 cases per year [1, 4].* Brucella* infection is diagnosed by using the Huddleston test and confirmed by the Wright test [5, 6].

We investigated the existence of clusters of brucellosis infected patients according to clinical presentation in Azerbaijan.

## 2. Materials and Methods

**Figure 1:**
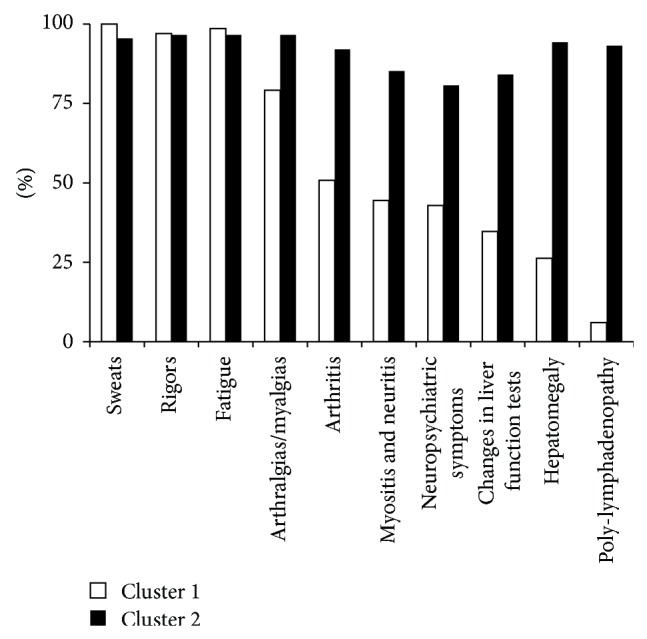
Percentage of symptoms by cluster groups. Note: changes in liver function tests include aminotransferase, aspartate aminotransferase, and gamma-glutamyl transferase; neuropsychiatric symptoms include depression, lack of concentration, and insomnia.

Brucellosis is a notifiable disease in Azerbaijan. In 2010, the Azerbaijan Ministry of Health initiated an official electronic surveillance system for all nationally notifiable diseases: the Electronic Integrated Disease Surveillance System (EIDSS) [7]. This computerized surveillance and reporting system integrates human, veterinary, and laboratory data from 158 sites across the country. This study used EIDSS data and included all brucellosis cases that reported fever (>38°C) for at least five days, at least 5 of the symptoms shown in Figure 1, and a confirmed test for* Brucella* spp. from July 2011 through July 2013.

We conducted a latent class cluster analysis (LCC) using Latent GOLD and R language to identify clusters [8]. LCC has been described in detail elsewhere [9]. Briefly, LCC uses the maximum likelihood estimation to classify cases according to the probabilities of all variables. This approach is better suited for binary data compared to traditional methods (e.g., hierarchical or *k*-means), which use some criterion of similarity or distance. Symptoms utilized in data analysis are shown in Figure 1. To determine the optimal cluster model, we used the Akaike's information criteria (AIC), the Bayesian information criteria (BIC), the model entropy measure, and the Vuong-Lo-Mendell-Rubin likelihood ratio test (VLMR). Lower AIC and BIC values suggest better fitting models. The entropy measures how well a model predicts classification; a value close to one indicates perfect classification [10]. The VLMR test compares a *k* cluster model with *k* − 1 cluster model.

## 3. Results

In total 454 brucellosis cases were included in the analysis. Seventy-three percent of cases were male, and the median age was 26 years. Over 34% of cases occurred during the March to May spring season. After fever, the three most common symptoms reported were sweats (99%), fatigue (98%), and rigors (97%). There was no correlation between the number of symptoms and age.

LCC analysis indicated that a two-cluster model was optimal. This model, compared to others with three, four, or five clusters, had the lowest AIC (4032.1) and BIC (4118.5) values and the entropy value (0.85) suggested a good certainty in model classification. The VLMR supported the two-cluster model (*P* value < 0.001). Cluster 1 had 366 cases and cluster 2 included 88 cases (Table 1). There were no significant differences between the two clusters in terms of age, gender, season of diagnosis, hospitalization, and reported contact with sick animals. However, patients in cluster 1 reported higher percentages of assistance in animal birth and consumption of unpasteurized products compared to patients in cluster 2.

Regarding symptoms, several differences were observed between the two clusters. With the exception of sweats, rigors, and fatigue, most symptoms were more reported among patients in cluster 2 (Figure 1). Patients in cluster 2 had higher percentages of poly-lymphadenopathy (93% versus 6%), hepatomegaly (84% versus 35%), arthritis (92% versus 51%), myositis and neuritis (85% versus 44%), changes in liver function tests (84% versus 35%), arthralgias/myalgias (97% versus 79%), and neuropsychiatric symptoms (81% versus 43%) compared to patients in cluster 1 (all *P* values < 0.001).

In an attempt to determine whether regional differences exist between the two clusters of patients, we examined the number of cases in regions at high risk for transmission: Imishli, Aghjabedi, Shamkir, and Beylagan. All cases from Imishli or Aghjabedi belonged to cluster 1; however, 7% and 76% of the cases from Shamkir and Beylagan belonged to cluster 2, respectively. Additionally, Beylagan was associated with cluster 2 (odds ratio = 11.1; 95% CI = 4.2–28.9; *P* value < 0.001).

## 4. Discussion

Using LCC and clinical presentation, we identified two clusters of brucellosis infection in Azerbaijan. Eighty-one percent of the cases were assigned to cluster 1 and 19% to cluster 2. In both clusters, fatigue, rigors, and night sweats were common. This finding was not surprising because fatigue is commonly associated with weakness and night sweats, and rigors appear commonly in late afternoon or evening when the body temperature rises [11].

We also observed differences in other clinical symptoms associated with brucellosis. Cases in cluster 2 had higher percentages of hepatomegaly, poly-lymphadenopathy, arthritis, myositis and neuritis, changes in liver function tests, and neuropsychiatric manifestations compared to cases in cluster 1. Lymphadenopathy is not a common feature of brucellosis and is more frequent in patients with severe disease [11]. Abnormal liver function tests are observed in acute cases and hepatomegaly is more likely to occur in patients with chronic brucellosis [12]. Musculoskeletal symptoms such as arthritis, arthralgias, myalgias, and myositis are frequent manifestations in brucellosis patients, with one-half of patients reporting significant morbidity [13]. Central nervous system involvement is uncommon, reported in 5% to 7% of all brucellosis cases [14].

The set of symptoms associated with the second cluster is not commonly reported and is observed in a subset of brucellosis patients [2, 11–16], indicating that patients in cluster 2 had a more severe illness. We also found the second cluster was associated with delays in seeking medical care. In comparison to patients in cluster 1, they sought medical care on average two weeks later (20.6 versus 31.6 days), suggesting that their symptoms got progressively worse, for instance, poly-lymphadenopathy. Additional research is required to understand the health-seeking factors of brucellosis cases.

We also found that 3 out of 4 cases reported in Beylagan region belonged to the second cluster. Beylagan, a region with a population of 86,000, located in south-central Azerbaijan near the border with Iran, is an endemic brucellosis area [1]. This region is focused on sheep and goat livestock production, and brucellosis in small ruminants is mainly caused by* Brucella melitensis*, which causes the most severe type of disease in humans [14], leading to the development of severe symptoms [2]. Therefore, our data suggest that Beylagan is a high-transmission area for this* Brucella* species. Available (and limited) subtyping data indicate that* B. melitensis* and* B. abortus* are responsible for human brucellosis in the county [17]. Research on species identification on human and animal samples from Beylagan will help explain the epidemiology of brucellosis.

Our study has limitations. First, consumption of unpasteurized products and assistance in animal birth are too broad questions that have multiple answers and do not allow the level of analysis desired. For instance, unpasteurized cheese is associated with* B. melitensis* and unpasteurized milk with* B. abortus*. Second, other symptoms related to brucellosis such as weakness, back pain, loss of appetite, anemia, and cough are not collected in EIDSS and, thus, were not analyzed. Despite these limitations, our analyses of surveillance data are useful to generate research hypothesis and provide baseline information for the development of health education programs to highlight the importance of early diagnosis and prompt treatment in the population.

In conclusion, based on the clinical spectrum of presenting symptoms, we identified two clusters of brucellosis in Azerbaijan. Patient in cluster 2 had a more severe clinical presentation and was associated with delays in seeking medical care. Most patients in cluster 2 were also from Beylagan, a region with high-density small livestock, suggesting a hot-spot area for* B. melitensis*. Future work needs to determine the factors that influence medical case seeking of brucellosis cases and identify brucellosis species.

## Figures and Tables

**Table 1:** Cluster analysis of symptoms of 454 brucellosis cases in Azerbaijan.

Feature	Total *n* = 454 (%)	Cluster 1 *n* = 366 (%)	Cluster 2 *n* = 88 (%)	*P* value
Age in years, median	26	26	27	0.983
Males	333 (74)	270 (74)	63 (72)	0.719
Diagnosis in spring season	124 (27)	100 (27)	24 (27)	0.993
Hospitalization	231 (55)	181 (53)	50 (60)	0.305
Assistance in animal birth	258 (57)	228 (62)	30 (34)	<0.001
Contact with sick animals	113 (25)	97 (26)	16 (18)	0.138
Consumption of unpasteurized products	173 (38)	155 (42)	18 (20)	<0.001
Number of symptoms, median [range]	6 [5–8]	6 [5–8]	9.5 [5–10]	<0.001
Days between disease onset and seeking medical care, mean	22.5	20.6	31.6	0.028

## Disclaimer

The views expressed herein are those of the authors and do not reflect the official policy or position of the Department of the Army, Department of Defense, or the US Government. Some of the authors are US government employees. This work was prepared as part of their official duties; there is no copyright to be transferred.
